# Supplementary material for: Modification of Mesenchymal Stem/Stromal Cell-Derived Small Extracellular Vesicles by Calcitonin Gene Related Peptide (CGRP) Antagonist: Potential Implications for Inflammation and Pain Reversal
Source: Cells. 2024 Mar 10;13(6):484. doi: 10.3390/cells13060484 (PMC10969778; doi:10.3390/cells13060484)
Supplement: Supplementary file 1 [file cells-13-00484-s001.zip › SUPPLEMENTARY TABLE S1.pdf]

Table S1. Human MSC exosome miRNA array

qPCR Primer	Mature miRNA name	Mature miRNA accession
HmiRQP0002	hsa-let-7a-5p	MIMAT0000062
HmiRQP0004	hsa-let-7b-5p	MIMAT0000063
HmiRQP0007	hsa-let-7d-5p	MIMAT0000065
HmiRQP0010	hsa-let-7e-5p	MIMAT0000066
HmiRQP0012	hsa-let-7f-5p	MIMAT0000067
HmiRQP0015	hsa-let-7g-5p	MIMAT0000414
HmiRQP0017	hsa-let-7i-5p	MIMAT0000415
HmiRQP0018	hsa-miR-100-5p	MIMAT0000098
HmiRQP0029	hsa-miR-106b-5p	MIMAT0000680
HmiRQP0030	hsa-miR-107	MIMAT0000104
HmiRQP0031	hsa-miR-10a	MIMAT0004555
HmiRQP0032	hsa-miR-10a-5p	MIMAT0000253
HmiRQP0034	hsa-miR-10b-5p	MIMAT0000254
HmiRQP3286	hsa-miR-1185-2-3p	MIMAT0022713
HmiRQP0056	hsa-miR-122-5p	MIMAT0000421
HmiRQP0078	hsa-miR-1246	MIMAT0005898
HmiRQP0087	hsa-miR-1255a	MIMAT0005906
HmiRQP0093	hsa-miR-125a	MIMAT0004602
HmiRQP0094	hsa-miR-125a-5p	MIMAT0000443
HmiRQP0096	hsa-miR-125b-5p	MIMAT0000423
HmiRQP0099	hsa-miR-126-3p	MIMAT0000445
HmiRQP0111	hsa-miR-127-3p	MIMAT0000446
HmiRQP0134	hsa-miR-1290	MIMAT0005880
HmiRQP0152	hsa-miR-1305	MIMAT0005893
HmiRQP0156	hsa-miR-130a-3p	MIMAT0000425
HmiRQP0160	hsa-miR-132	MIMAT0004594
HmiRQP0161	hsa-miR-132-3p	MIMAT0000426
HmiRQP0173	hsa-miR-136-5p	MIMAT0000448
HmiRQP0175	hsa-miR-137	MIMAT0000429
HmiRQP0181	hsa-miR-140-5p	MIMAT0000431
HmiRQP0186	hsa-miR-142-3p	MIMAT0000434
HmiRQP0188	hsa-miR-143-3p	MIMAT0000435
HmiRQP0190	hsa-miR-144-3p	MIMAT0000436
HmiRQP0192	hsa-miR-145-5p	MIMAT0000437
HmiRQP0195	hsa-miR-146a	MIMAT0004608
HmiRQP0196	hsa-miR-146a-5p	MIMAT0000449
HmiRQP0204	hsa-miR-148a-3p	MIMAT0000243
HmiRQP0206	hsa-miR-148b-3p	MIMAT0000759
HmiRQP0208	hsa-miR-149-5p	MIMAT0000450
HmiRQP0210	hsa-miR-150-5p	MIMAT0000451
HmiRQP0211	hsa-miR-151a-3p	MIMAT0000757
HmiRQP0219	hsa-miR-154-5p	MIMAT0000452
HmiRQP0222	hsa-miR-15a	MIMAT0004488
HmiRQP0223	hsa-miR-15a-5p	MIMAT0000068
HmiRQP0225	hsa-miR-15b-5p	MIMAT0000417
HmiRQP0227	hsa-miR-16-5p	MIMAT0000069
HmiRQP0233	hsa-miR-181a-2-3p	MIMAT0004558
HmiRQP0231	hsa-miR-181a-3p	MIMAT0000270
HmiRQP0232	hsa-miR-181a-5p	MIMAT0000256
HmiRQP0238	hsa-miR-182-3p	MIMAT0000260

Table S1. Human MSC exosome miRNA array

HmiRQP0245	hsa-miR-184	MIMAT0000454
HmiRQP0247	hsa-miR-185-5p	MIMAT0000455
HmiRQP0251	hsa-miR-187-3p	MIMAT0000262
HmiRQP0255	hsa-miR-18a-5p	MIMAT0000072
HmiRQP0263	hsa-miR-191-5p	MIMAT0000440
HmiRQP0277	hsa-miR-193a-5p	MIMAT0004614
HmiRQP0279	hsa-miR-193b-5p	MIMAT0004767
HmiRQP0284	hsa-miR-196a-5p	MIMAT0000226
HmiRQP0286	hsa-miR-196b-5p	MIMAT0001080
HmiRQP3038	hsa-miR-197-5p	MIMAT0022691
HmiRQP0289	hsa-miR-199a-3p	MIMAT0000232
HmiRQP0290	hsa-miR-199a-5p	MIMAT0000231
HmiRQP0289	hsa-miR-199b-3p	MIMAT0004563
HmiRQP0291	hsa-miR-199b-5p	MIMAT0000263
HmiRQP0293	hsa-miR-19a-3p	MIMAT0000073
HmiRQP0295	hsa-miR-19b-3p	MIMAT0000074
HmiRQP0298	hsa-miR-200a-3p	MIMAT0000682
HmiRQP0307	hsa-miR-205-5p	MIMAT0000266
HmiRQP0312	hsa-miR-20a-5p	MIMAT0000075
HmiRQP0314	hsa-miR-20b-5p	MIMAT0001413
HmiRQP0320	hsa-miR-214-3p	MIMAT0000271
HmiRQP0316	hsa-miR-21-5p	MIMAT0000076
HmiRQP0327	hsa-miR-218-5p	MIMAT0000275
HmiRQP0338	hsa-miR-221-3p	MIMAT0000278
HmiRQP0339	hsa-miR-222	MIMAT0000279
HmiRQP0339	hsa-miR-222-3p	MIMAT0000279
HmiRQP0342	hsa-miR-223-3p	MIMAT0000280
HmiRQP0332	hsa-miR-22-3p	MIMAT0000077
HmiRQP0344	hsa-miR-23a-3p	MIMAT0000078
HmiRQP0346	hsa-miR-23b-3p	MIMAT0000418
HmiRQP0349	hsa-miR-24-3p	MIMAT0000080
HmiRQP0352	hsa-miR-25-3p	MIMAT0000081
HmiRQP0354	hsa-miR-26a-5p	MIMAT0000082
HmiRQP0357	hsa-miR-26b-5p	MIMAT0000083
HmiRQP0361	hsa-miR-27b-3p	MIMAT0000419
HmiRQP0362	hsa-miR-28-5p	MIMAT0000085
HmiRQP0364	hsa-miR-296-5p	MIMAT0000690
HmiRQP0369	hsa-miR-299-5p	MIMAT0002890
HmiRQP0371	hsa-miR-29a-3p	MIMAT0000086
HmiRQP0373	hsa-miR-29b-3p	MIMAT0000100
HmiRQP0375	hsa-miR-29c-3p	MIMAT0000681
HmiRQP0378	hsa-miR-301a-3p	MIMAT0000688
HmiRQP1518	hsa-miR-3065-5p	MIMAT0015066
HmiRQP0391	hsa-miR-30a-5p	MIMAT0000087
HmiRQP0398	hsa-miR-30d-5p	MIMAT0000245
HmiRQP0400	hsa-miR-30e-5p	MIMAT0000692
HmiRQP1523	hsa-miR-320e	MIMAT0015072
HmiRQP0410	hsa-miR-323a-3p	MIMAT0000755
HmiRQP0404	hsa-miR-32-5p	MIMAT0000090
HmiRQP0421	hsa-miR-335-5p	MIMAT0000765
HmiRQP0423	hsa-miR-337-3p	MIMAT0000754

Table S1. Human MSC exosome miRNA array

HmiRQP0424	hsa-miR-337-5p	MIMAT0004695
HmiRQP0436	hsa-miR-342-3p	MIMAT0000753
HmiRQP0440	hsa-miR-34a-5p	MIMAT0000255
HmiRQP1907	hsa-miR-3613-3p	MIMAT0017991
HmiRQP0446	hsa-miR-361-5p	MIMAT0000703
HmiRQP0448	hsa-miR-362-5p	MIMAT0000705
HmiRQP0449	hsa-miR-363-3p	MIMAT0000707
HmiRQP0451	hsa-miR-365a-3p	MIMAT0000710
HmiRQP1944	hsa-miR-3665	MIMAT0018087
HmiRQP0454	hsa-miR-369-3p	MIMAT0000721
HmiRQP0463	hsa-miR-374a-5p	MIMAT0000727
HmiRQP0464	hsa-miR-374b-5p	MIMAT0004955
HmiRQP0467	hsa-miR-376a-3p	MIMAT0000729
HmiRQP0472	hsa-miR-377-3p	MIMAT0000730
HmiRQP2046	hsa-miR-378g	MIMAT0018937
HmiRQP2182	hsa-miR-378i	MIMAT0019074
HmiRQP0476	hsa-miR-379-5p	MIMAT0000733
HmiRQP0480	hsa-miR-382-5p	MIMAT0000737
HmiRQP0484	hsa-miR-409-3p	MIMAT0001639
HmiRQP0487	hsa-miR-411-3p	MIMAT0004813
HmiRQP0486	hsa-miR-411-5p	MIMAT0003329
HmiRQP0492	hsa-miR-423-5p	MIMAT0004748
HmiRQP0494	hsa-miR-424-5p	MIMAT0001341
HmiRQP1612	hsa-miR-4286	MIMAT0016916
HmiRQP0501	hsa-miR-432-5p	MIMAT0002814
HmiRQP2084	hsa-miR-4454	MIMAT0018976
HmiRQP2100	hsa-miR-4466	MIMAT0018993
HmiRQP0503	hsa-miR-448	MIMAT0001532
HmiRQP2128	hsa-miR-4488	MIMAT0019022
HmiRQP0506	hsa-miR-450a-5p	MIMAT0001545
HmiRQP2159	hsa-miR-4516	MIMAT0019053
HmiRQP0509	hsa-miR-451a	MIMAT0001631
HmiRQP0516	hsa-miR-455-5p	MIMAT0003150
HmiRQP2241	hsa-miR-4647	MIMAT0019709
HmiRQP2495	hsa-miR-4792	MIMAT0019964
HmiRQP0540	hsa-miR-497-5p	MIMAT0002820
HmiRQP0543	hsa-miR-499a-5p	MIMAT0002870
HmiRQP1537	hsa-miR-514b-5p	MIMAT0015087
HmiRQP0610	hsa-miR-525-5p	MIMAT0002838
HmiRQP0616	hsa-miR-532-3p	MIMAT0004780
HmiRQP0622	hsa-miR-543	MIMAT0004954
HmiRQP0627	hsa-miR-548a-5p	MIMAT0004803
HmiRQP2631	hsa-miR-548ar-3p	MIMAT0022266
HmiRQP2015	hsa-miR-548y	MIMAT0018354
HmiRQP0673	hsa-miR-574-3p	MIMAT0003239
HmiRQP0674	hsa-miR-574-5p	MIMAT0004795
HmiRQP0675	hsa-miR-575	MIMAT0003240
HmiRQP0684	hsa-miR-582-5p	MIMAT0003247
HmiRQP2775	hsa-miR-6089	MIMAT0023714
HmiRQP0716	hsa-miR-612	MIMAT0003280
HmiRQP0720	hsa-miR-615-3p	MIMAT0003283

Table S1. Human MSC exosome miRNA array

HmiRQP0734	hsa-miR-626	MIMAT0003295
HmiRQP0740	hsa-miR-630	MIMAT0003299
HmiRQP0760	hsa-miR-650	MIMAT0003320
HmiRQP0769	hsa-miR-658	MIMAT0003336
HmiRQP0786	hsa-miR-7-5p	MIMAT0000252
HmiRQP0796	hsa-miR-767-5p	MIMAT0003882
HmiRQP4563	hsa-miR-7975	MIMAT0031178
HmiRQP0802	hsa-miR-802	MIMAT0004185
HmiRQP0817	hsa-miR-888-5p	MIMAT0004916
HmiRQP0837	hsa-miR-93-5p	MIMAT0000093
HmiRQP0853	hsa-miR-98-5p	MIMAT0000096
HmiRQP0854	hsa-miR-99a-5p	MIMAT0000097
HmiRQP0856	hsa-miR-99b	MIMAT0004678
HmiRQP0857	hsa-miR-99b-5p	MIMAT0000689
HK1(RNU6-2)	HK1(RNU6-2)	NR_002752
HK2(SNORD44)	HK2(SNORD44)	NR_002750
HK3(SNORD48)	HK3(SNORD48)	NR_002745
HK4(SNORD47)	HK4(SNORD47)	NR_002746
HK5(SNORD49A)	HK5(SNORD49A)	NR_002744
HK6(SNORD68)	HK6(SNORD68)	NR_002450
