# Supplementary material for: Modification of Mesenchymal Stem/Stromal Cell-Derived Small Extracellular Vesicles by Calcitonin Gene Related Peptide (CGRP) Antagonist: Potential Implications for Inflammation and Pain Reversal
Source: Cells. 2024 Mar 10;13(6):484. doi: 10.3390/cells13060484 (PMC10969778; doi:10.3390/cells13060484)
Supplement: Supplementary file 1 [file cells-13-00484-s001.zip › SUPPLEMENTARY TABLE S2.pdf]

Table S2. Cytokines and growth factors array

**Protein name**	
Eotaxin	AR
Eotaxin-2	βFGF
ICAM-1	βNGF
IFN-γ	EGF
I-309	EGF R
IL-1a	FGF-4
IL-1β	FGF-6
IL-2	FGF-7
IL-3	G-CSF
IL-4	GDNF
IL-6	GM-CSF
IL-6 sR	HB-EGF
IL-7	HGF
IL8	IGFBP-1
IL-10	IGFBP-2
IL-11	IGFBP-3
IL12-p40	IGFBP-4
IL12-p70	IGFBP-6
IL-13	IGF-I
IL-15	IGF-I SR
IL-16	IGF-II
IL17	M-CSF
IP-10	MCSF R
MCP-1	NT-3
MCP-2	NT-4
MIG	PDGF Rα
MIP-1-α	PDGF Rβ
MIP-1-β	PDGF-AA
MIP-1-δ	PDGF-AB
RANTES	PDGF-BB
TNF-α	PIGF
TNF-β	SCF
sTNF-RI	SCF R
sTNF RII	TGF-α
TIMP-2	TGF-β
	TGF-β2
	TGF-β3
	VEGF
	VEGF R2
	VEGF R3
	VEGF D
