# Supplementary material for: Modification of Mesenchymal Stem/Stromal Cell-Derived Small Extracellular Vesicles by Calcitonin Gene Related Peptide (CGRP) Antagonist: Potential Implications for Inflammation and Pain Reversal
Source: Cells. 2024 Mar 10;13(6):484. doi: 10.3390/cells13060484 (PMC10969778; doi:10.3390/cells13060484)
Supplement: Supplementary file 1 [file cells-13-00484-s001.zip › SUPPLEMENTARY TABLE S3.pdf]

Table S3. Human Macrophage Polarization Marker qPCR Array

Gene name	
BMP7	TLR2
HIF1A	NONO
IL1B	CD68
MRC1	HLA-DRB1
STAT6	IL6
ACTB	PPARG
CCL2	TLR4
HLA-DQA1	PPIH
IL1R1	CD80
NFKB1	IFNG
TGFB1	IRF4
GAPDH	SOCS3
CD163	TLR8
HLA-DQB1	GDC
IL1R2	CD86
NOS2	IL10
TLR1	KLF4
LDHA	STAT1
CD200R1	TNF
HLA-DRA	PPC
IL23A	FABP4
PECAM1	IL12A
STAT3	MMP9
VEGFA	
