# Supplementary material for: Modification of Mesenchymal Stem/Stromal Cell-Derived Small Extracellular Vesicles by Calcitonin Gene Related Peptide (CGRP) Antagonist: Potential Implications for Inflammation and Pain Reversal
Source: Cells. 2024 Mar 10;13(6):484. doi: 10.3390/cells13060484 (PMC10969778; doi:10.3390/cells13060484)
Supplement: Supplementary file 1 [file cells-13-00484-s001.zip › SUPPLEMENTARY TABLE S4.pdf]

Table S4. RT2 Profiler neuropathic & inflammatory array

**Gene name**	
ACE	MAPK1
ADORA1	MAPK14
ADRB2	MAPK3
ALOX5	MAPK8
BDKRB1	NGF
BDNF	NTRK1
CACBA1B	OPRD1
CALCA	OPRK1
CCK	OPRM1
CCKBR	P2RX3
CCL2	P2RX4
CCRS	P2RX7
CD200	P2RXY1
CD4	POYN
CHRN4	PENK
CNR1	PLA2G1B
CNR2	PNOC
COMT	PROK2
CSF1	PTGER1
CX3CR1	PTGER3
DBH	PTGER4
EDN1	PTGES
EDNRA	PTGES2
FAAH	PTGES3
GCH1	PTGS1
GDNF	PTGS2
GRIN1	SCN10A
GRIN2B	SCN11A
GRM1	SCN3A
GRM5	SCN9A
HTR1A	SLC6A2
HTR2A	TAC1
IL10	TACR1
LI18	TLR2
LI1A	TLR4
IL1B	TNF
IL2	TRPA1
IL6	TRPV1
ITGAM	TRPV3
ITGB2	ACTB
KCNIP3	B2M
KCNJ6	GAPDH
KCNQ2	HRPT1
KCNQ3	RPLP0
MAOB	HGDC
